# An original aneuploidy-related gene model for predicting lung adenocarcinoma survival and guiding therapy

**DOI:** 10.1038/s41598-024-58020-y

**Published:** 2024-04-07

**Authors:** Yalei Zhang, Dongmei Li

**Affiliations:** Department of Thoracic Oncology, The First Affiliated Hospital of Guangzhou Medical University, Guangzhou, 510032 China

**Keywords:** Lung adenocarcinoma, Aneuploidy, Riskscore model, Nomogram, Therapy, Lung cancer, Cancer, Surgical oncology

## Abstract

Aneuploidy is a hallmark of cancers, but the role of aneuploidy-related genes in lung adenocarcinoma (LUAD) and their prognostic value remain elusive. Gene expression and copy number variation (CNV) data were enrolled from TCGA and GEO database. Consistency clustering analysis was performed for molecular cluster. Tumor microenvironment was assessed by the xCell and ESTIMATE algorithm. Limma package was used for selecting differentially expressed genes (DEGs). LASSO and stepwise multivariate Cox regression analysis were used to establish an aneuploidy-related riskscore (ARS) signature. GDSC database was conducted to predict drug sensitivity. A nomogram was designed by rms R package. TCGA-LUAD patients were stratified into 3 clusters based on CNV data. The C1 cluster displayed the optimal survival advantage and highest inflammatory infiltration. Based on integrated intersecting DEGs, we constructed a 6-gene ARS model, which showed effective prediction for patient’s survival. Drug sensitivity test predicted possible sensitive drugs in two risk groups. Additionally, the nomogram exhibited great predictive clinical treatment benefits. We established a 6-gene aneuploidy-related signature that could effectively predict the survival and therapy for LUAD patients. Additionally, the ARS model and nomogram could offer guidance for the preoperative estimation and postoperative therapy of LUAD.

## Introduction

Lung cancer now ranks the top two major causes of cancer-relevant mortalities in both sexes all over the world^1^. The latest data showed that global new lung cancer deaths in 2020 was close to 1.8 million^1^. Lung adenocarcinoma (LUAD), which is the predominate type of lung cancer^2,3^, has witnessed an increase in incidence in the last 15 years and become the most infiltrative form of lung cancer^4,5^. For LUAD patients, the treatment results are far from satisfactory due to the delay in diagnosis and limitations of traditional therapies^6^. Hence, discovering novel biomarkers and individualized prognosis are urgently needed to improve early detection and treatment of LUAD patients.

Aneuploidy, also known as somatic cell copy number alterations, is widely detected in human tumors and has been considered as the cause of tumorigenesis^7^. Researchs on prostate cancer patients and head and neck squamous cell carcinomas pointed out that the increase of tumor aneuploidy contributed to a higher risk of fatal diseases^8,9^. The research on aneuploidy has also expanded to the field of lung cancer. Gao B’s team descripted a landscape of chromosome arm aneuploidy in LUAD in detail^10^. In non-small cell lung cancer (NSCLC) patients undergoing radiotherapy, aneuploidy was reported to cooperate with mutational burden for survival evaluation^11^. Spurr LF et al.^12^ proposed that aneuploidy in cancer could help predict survival after immunotherapy in various cancers. These results indicated that aneuploidy may be a useful biomarker for tumor immunotherapy. In addition, copy number variation (CNV) is a polymorphism found in the human genome that primarily involves DNA segments larger than 1 kb. It has been reported that in cancer cells, chromosomal aneuploidy can lead to copy number alterations^13^. Hu et al.^14^ identified a total of nine genes to be able to independently predict the prognosis of breast cancer patients based on public databases of breast cancer and CNV data. Bian et al.^15^ comprehensively analyzed CNV differential data and differentially expressed gene data from TCGA and screened eight CNV driver genes (including *AKR1B15*, *TRIM16L*, *CBX2*, *CDCA8*, *EZH2*, *FLVCR1*, *EPS8L3*, and *GPRIN1*) to generate a prognostic model that could well predict the prognosis of patients with hepatocellular carcinoma. In particular, there are studies on cancer biomarkers based on screening of aneuploidy-related genes that remain unclear. In addition, there is a lack of models for indicating the efficacy of immunotherapy or prognosis in LUAD based on aneuploidy.

To our knowledge, it is only the first time that risk modeling based on aneuploidy-related genes and screening of key genes to predict patient prognosis have been performed in LUAD. Firstly, consensus clustering^16^ was applied to classify different patient subgroups using the CNV data from TCGA database. Then, after intersecting DEGs between subgroups for WGCNA^17^ and aneuploidy score related genes, LASSO analysis^18^ was performed. Finally, 6 genes were selected and an aneuploidy-related model was constructed to guide survival prediction and therapy selection for LUAD patients.

## Material and methods

### Ethics statement

Data in our study were downloaded from online databases without any in vitro or in vivo tests.

### Study source

The latest expression data and clinical follow-up information of 387 LUAD samples were downloaded from the TCGA database (https://cancergenome.nih.gov, access date: June 9, 2023) as the testing cohort. The GSE31210 dataset with clinical survival information was retrieved from the Gene Expression Omnibus (GEO, https://www.ncbi.nlm.nih.gov/geo/, access date: June 9, 2023) website as the validation cohort. Genomic aneuploidy score for TCGA-LUAD samples (Table S1) was derived from an article^19^. Additionally, two immunotherapeutic datasets with anti- programmed cell death 1 (*PD-1*) checkpoint inhibition therapy GSE78220^20^ and GSE135222^21,22^ were selected from the GEO database. It is worth mentioning, this study aimed to predict prognosis, immunotherapy response in LUAD samples, thus didn’t need healthy individuals.

### Cluster analysis

Based on the copy number variation data from the TCGA database, GISTIC 2.0 software (version 6.15.28, https://cloud.genepattern.org, refgene file = Human_Hg19.mat, focal length cutoff = 0.50, gene gistic = yes, confidence level = 0.9. Other parameters were set as default)^23^ was used to analyze amplification and deletion regions using TCGA-LUAD data. Then, consistency clustering analysis was conducted. The ConsensusClusterPlus R package (parameters: maxK = 5, reps = 100, pItem = 0.8, pFeature = 1, clusterAlg = “kmdist”, distance = “pearson”)^16^ was implemented. The optimal number of clusters was determined by cumulative distribution function (CDF) and CDF Delta area curve.

### Evaluation of immune cell infiltration in different clusters

The xCell tool offers 64 cell types, including immune cells, stromal cells, stem cells, and other cells. Therefore, the xCell algorithm was used to calculate the scores for 64 cell types in the xCell R package (xCellAnalysis function run with the ‘rnaseq = TRUE’ option)^24^. For supplement, the sum of immune and stromal scores was computed through ESTIMATE R package^25^.

### Identification of differentially expressed genes in clusters

Differential gene analysis was conducted applying the R package “limma”^26^ for distinguishing the DEGs between different clusters. Filtering criteria was set at log2 fold change |log2FC|> log2 (1.2) and false discovery rate (FDR) < 0.05 using BenjaminiHochberg correction^27^. Volcano and Venn plots were employed to display the results.

### Co-expression network construction

WGCNA can gather genes and recruit modules through analogous gene expression patterns and investigate the correlation between modules and particular characteristics (clinic pathologic feature of patients, etc.)^28^. Hence, we applied the R package “WGCNA”^17^ to generate a scale-free co-expression network using the obtained DEGs.

### Establishment of a prognostic risk model for LUAD patients

Based on aneuploidy-related key module genes, Univariate cox regression analysis was conducted to screen genes relevant to LUAD prognosis. Subsequently, glmnet in the R software package (parameters: alpha = 1 and nlambda = 100)^29^ was used for LASSO Cox regression analysis, followed by stepwise multivariate Cox regression analysis. The aneuploidy-related gene scores (ARS) was calculated based on the following formula (1):

1 $$ ARS = \sum \beta i \times {\text{Exp }}i $$

The βi here means the coefficient value of selected gene, and Exp i means the expression level of selected gene.

The surv_cutpoint function in survminer package^30^ was adopted to distinguish the optimal point to separate LUAD patients into high and low ARS groups.

### Construction of a nomogram and validation

The independent indicators such as ARS and clinical features were used to design a nomogram applying “rms” R package (parameters ‘lp = F, maxscale = 100, fun.at = c(1,0.8,0.6,0.4,0.2,0)’ for ‘nomogram’)^31^ in TCGA-LUAD cohort. Calibration curves were plotted to evaluate the consistency of the model between the ideal and actual status. The clinical practicality of the nomogram was also evaluated adopting decision curve.

### LUAD cell line and drug sensitivity prediction

Drug sensitivity data concerning LUAD cell lines were downloaded from Genomics of Drug Sensitivity in Cancer (GDSC) database^32^. The antitumor drug area under concentration–time curve (AUC) was employed as the drug response index, Spearman correlation analysis was conducted to compute the relevance between AUC and ARS. |Rs|> 0.35 and FDR < 0.05 were defined as noticeably relevant. At the same time, we also analyzed the drug sensitivity in all the risk groups. The AUC values of LUAD cell lines were collected from The Cancer Cell Line Encyclopedia (CCLE) database^33^ and correlation and difference analysis were performed.

### Statistical analysis

All statistical analyses were performed using R software (version 4.0.3). The prognostic differences were displayed through Kaplan–Meier curves along with log-rank test. Receiver operator characteristic (ROC) curves were drawn using “timeROC” package (cause = 1, weighting = “marginal”, times = c(1,3,5) and iid = TRUE)^34^. Moreover, Sangerbox^35^ (http://sangerbox.com/home.html) was used for data processing in this research. Statistical significance was defined at *p* value < 0.05.

## Results

### Identification of molecular subtypes based on CNV data

**Figure 1:**
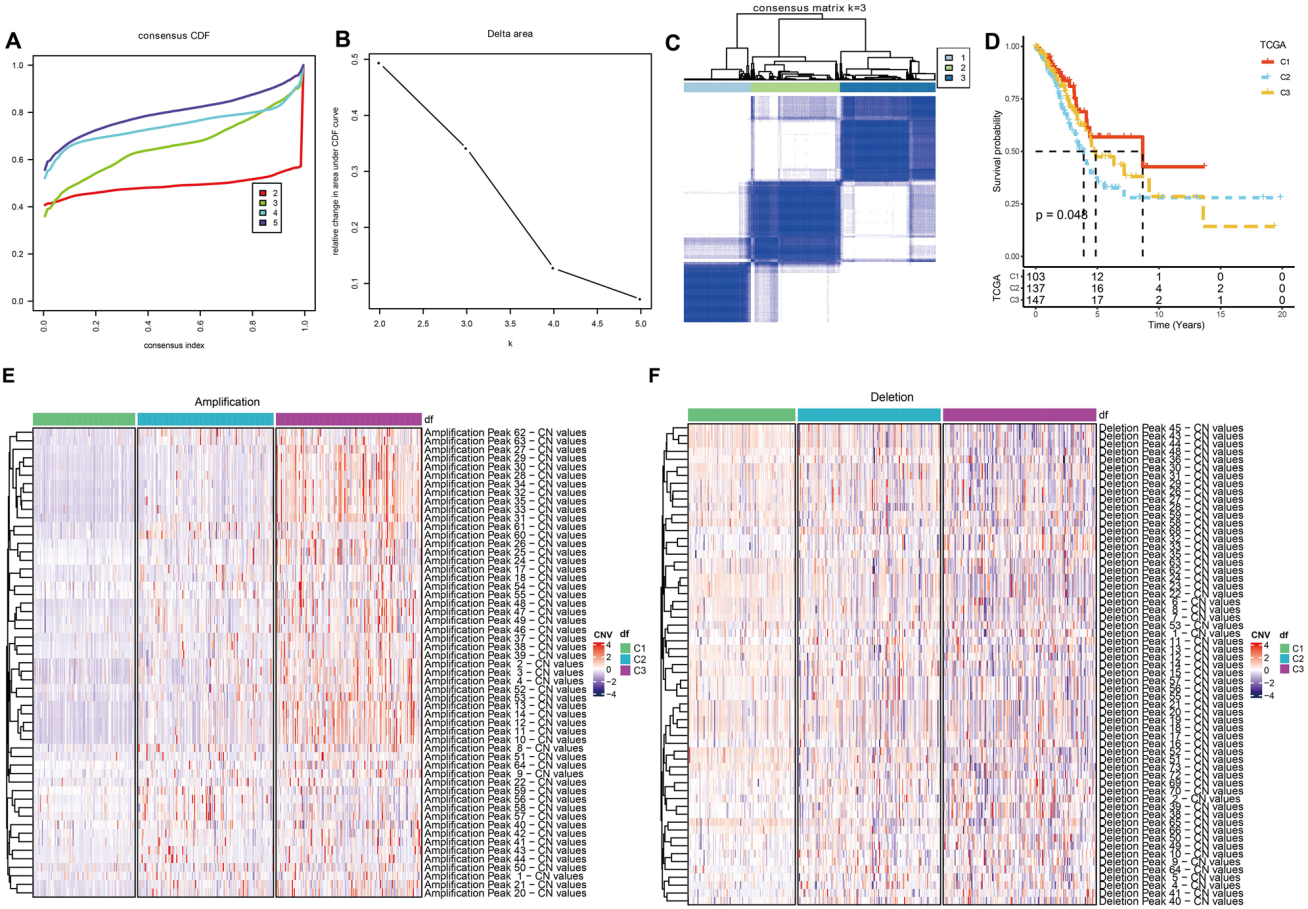
Identification of molecular subtypes based on CNV data. (**A**) CDF curve from k = 2–5. (**B**) CDF Delta area curve when k = 2–5. (**C**) A clustering heatmap when k = 3 in TCGA-LUAD cohort. (**D**) Kaplan–Meier survival analysis. (**E**) Amplification regions in three clusters. (**F**) Deletion regions in three clusters.

**Figure 2:**
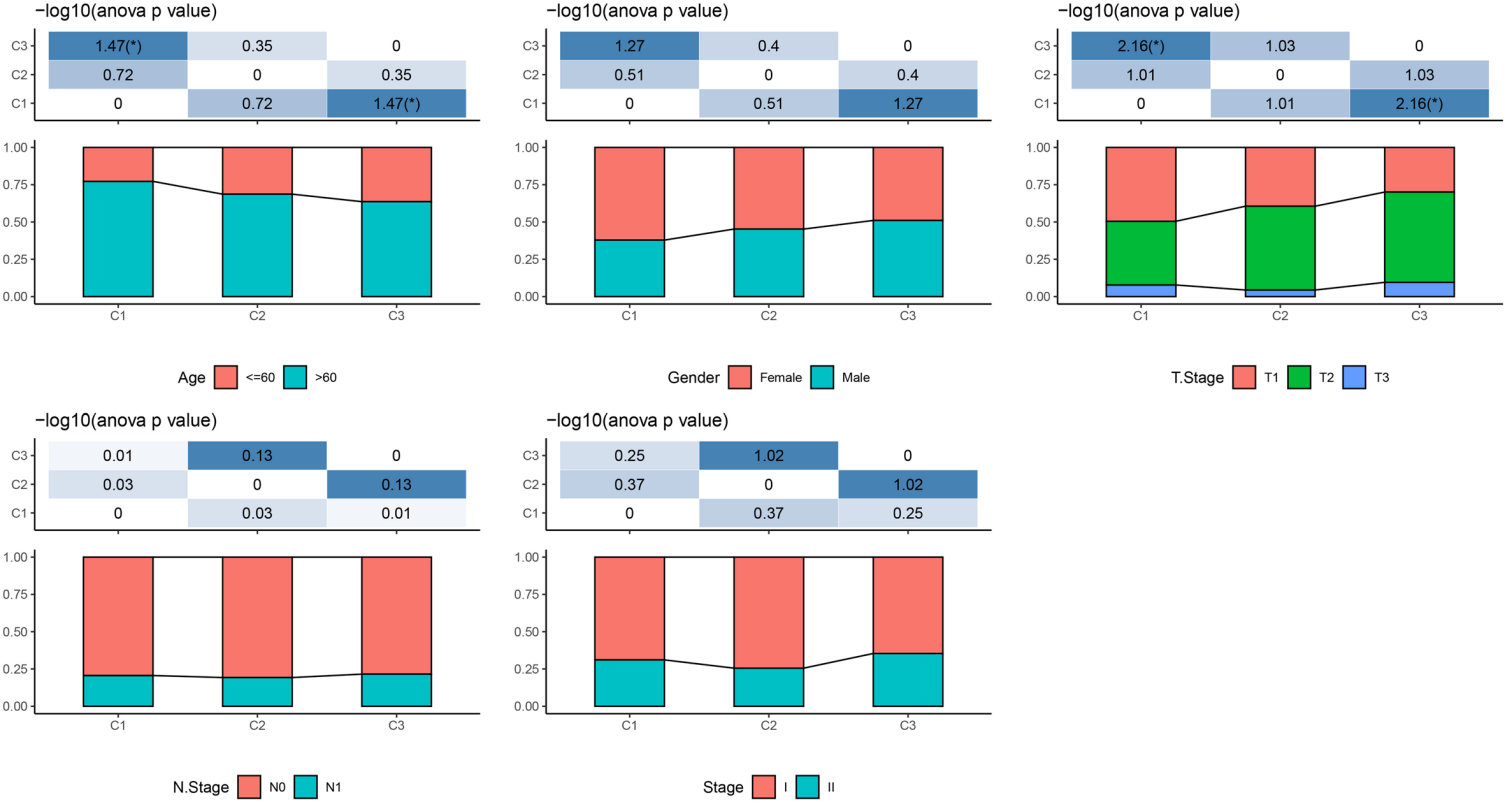
The distribution of clinical features in 3 clusters. The distribution of clinical features, such as Gender, T stage, N stage, Stage and Age, in 3 clusters.

The CDF and CDF Delta area curves showed that a stable clustering result was obtained when cluster number k is selected as 3 (Fig. 1A,B). The clustering TCGA-LUAD samples in a clustering heatmap displayed clear boundaries among three molecular subtypes (Fig. 1C). Meanwhile, the survival analysis demonstrated that the C1 subtype exhibited a longer survival time compared to the C2 and C3 subtypes (Fig. 1D). The amplification and deletion regions in the three clusters were shown in heatmaps (Fig. 1E,F). We found that the C1 subgroup with the best prognosis exhibited the least gene amplification and deletion. Finally, analysis on the distribution of different clinical characteristics in three subtypes demonstrated that C1 subtypes was characterized by more female, age over 60 years, and more patients in T1 early stage (Fig. 2).

### C1 subtypes with better prognosis exhibited higher level of immune infiltration

**Figure 3:**
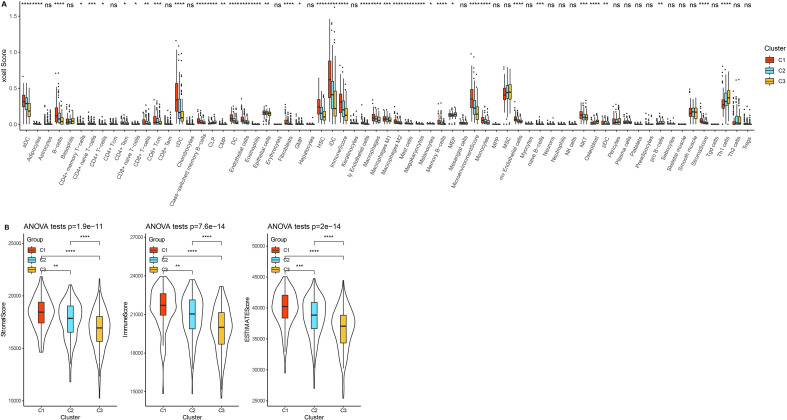
Immune characteristics in three subtypes. (**A**) Xcell score for assessing the combined level of immune cell and stromal cell types. (**B**) EXTIMATE analysis. *p < 0.05, **p < 0.01, ***p < 0.001, ***p < 0.0001, ns: no significance.

Subsequently, we analyzed the immune infiltration status among the three subtypes. Xcell algorithm revealed that the higher scores of Dendritic cells (DC), activated dendritic cells (aDC), conventional dendritic cells (cDC), immature dendritic cells (iDC), plasmacytoid dendritic cells (pDC), B cells, CD8 + T cell, CD8 + Central Memory T cell, endothelial cells, epithelial cells, fibroblasts, macrophages, macrophages M1, macrophages M2, immunescore and microenvironmentscore were enriched in C1 subtypes (Fig. 3A). ESTIMATE analysis further supported above finding, as C1 subtypes had the highest Stromal Score, Immune Score and ESTIMATE Score among 3 clusters (Fig. 3B).

### Screening of differentially expressed genes

**Figure 4:**
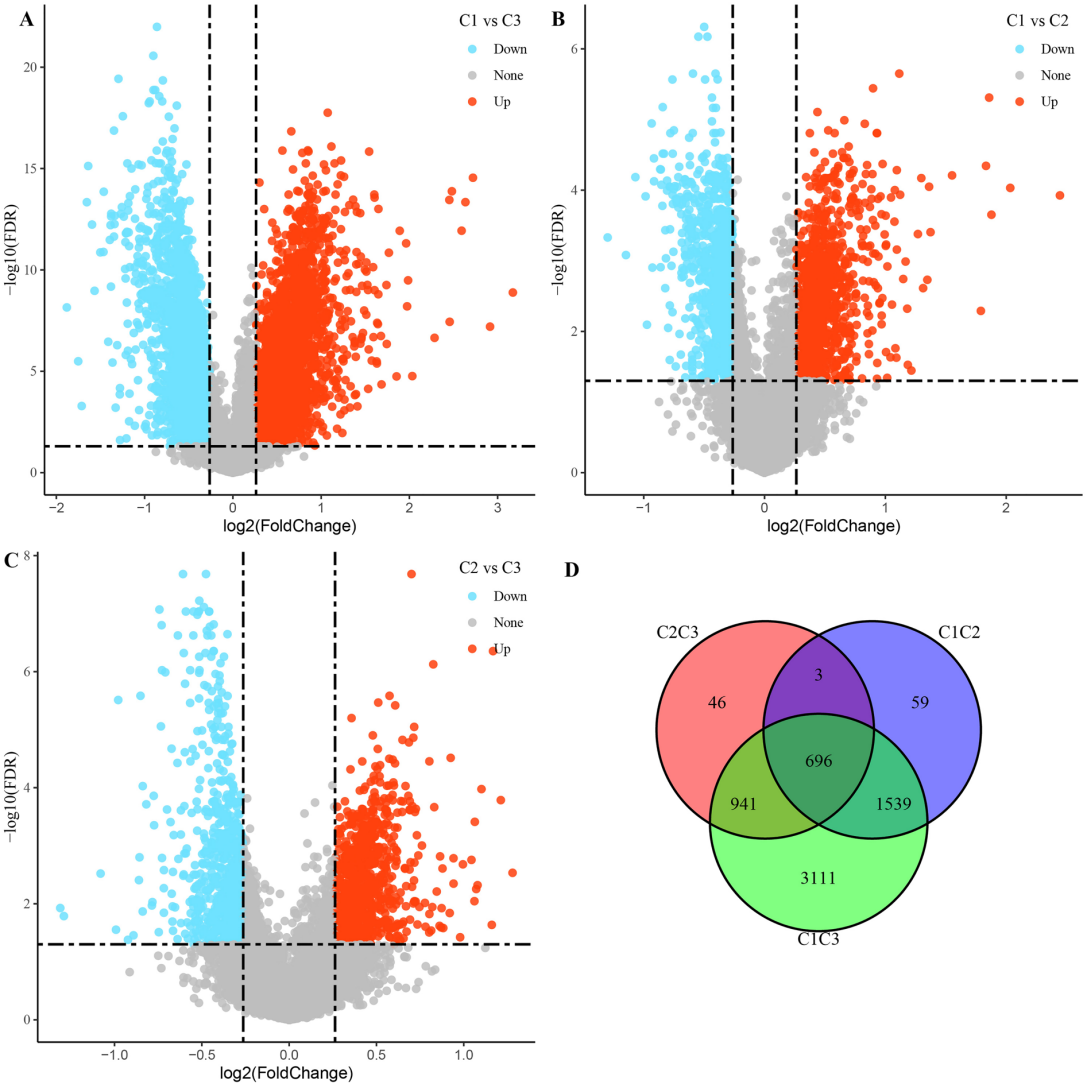
Differentially expressed genes analysis among 3 clusters. (**A**–**C**) Volcano plot depicting DEGs among 3 clusters. (**D**) Venn diagram showing the intersection of DEGs among 3 clusters.

As displayed in Fig. 4A–C. The most 6287 DEGs (3048 up-regulated and 3239 down-regulated) were identified in C1 and C3 clusters. 2297 DEGs (1267 up-regulated and 1030 down-regulated) were identified in the C1 and C2 clusters. 1686 DEGs (968 up-regulated and 718 down-regulated) were identified in the C2 and C3 clusters. By integrating three groups of DEGs, we obtained a total of 696 common DEGs (Fig. 4D). Functional enrichment analysis to further understand the differences in gene and functional levels between clusters. Overall, immune-related pathways were activated in the C1 subtype, and cell cycle-related pathways were activated in C2 and C3 (Supplementary Fig. 1A–C).

### Identification of key modules genes based on co-expression network

**Figure 5:**
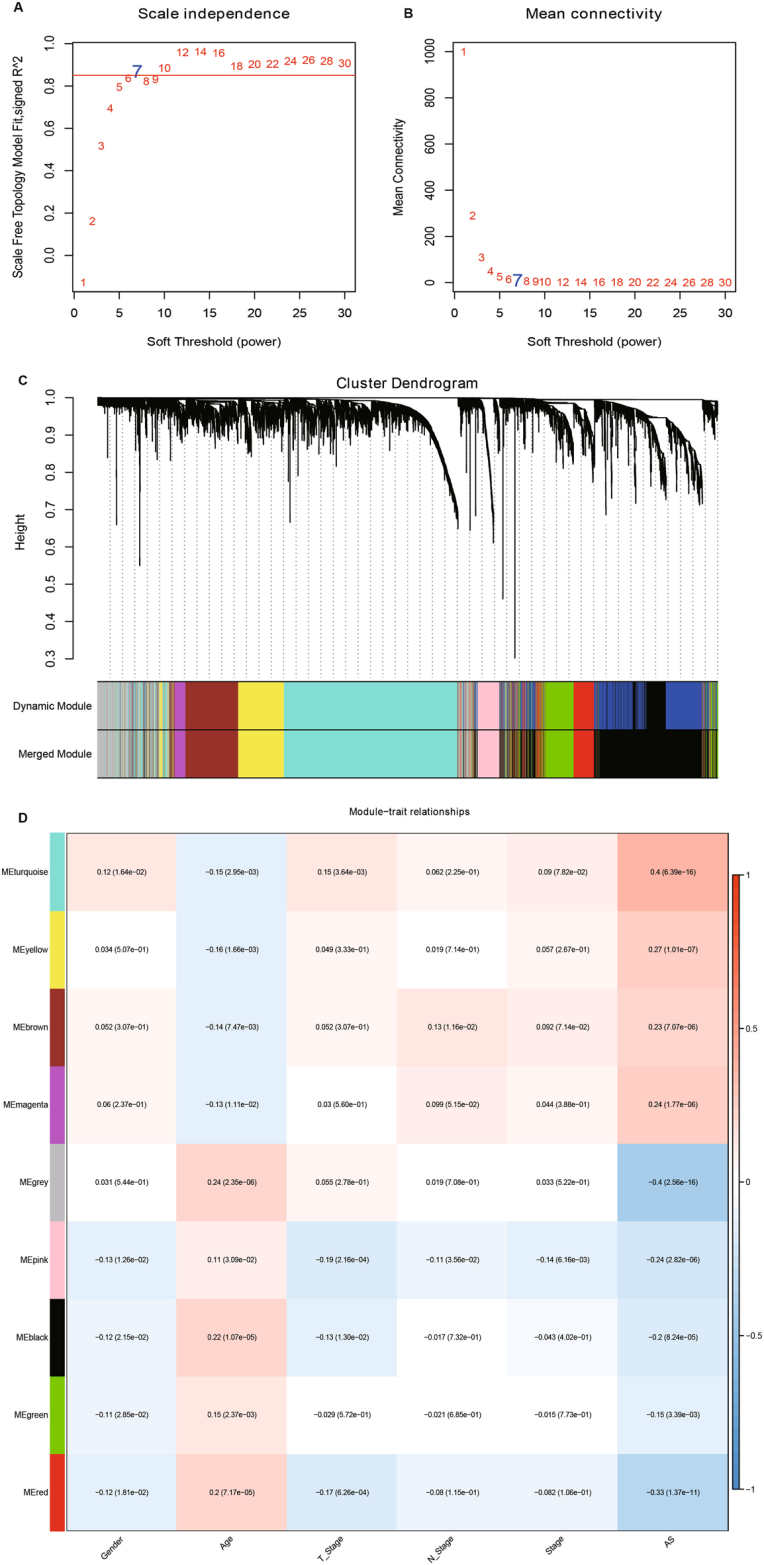
Co-expression network construction and identification of key modules. (**A**,**B**) Screening of soft thresholds and the relationship between soft thresholds and connectivity. (**C**) Building a hierarchical clustering tree. (**D**) Correlation analysis of 9 modules with clinical information and aneuploidy score (the correlation coefficient and *p* value were filled in each intersecting grid). The grey modules are collections of genes that cannot be aggregated to other modules.

We based our data on 387 expression profiles in the TCGA-LUAD database and proposed differentially expressed genes from them. When the correlation coefficient is greater than 0.9, the optimal soft threshold is set to 7 to screen for co-expressed modules. To ensure that the network is scale-free, we set β to 12 and convert the expression matrix to a topology matrix. Following the criteria for hybrid dynamic shear trees, the number of genes per gene network module was set to a minimum of 30. (Fig. 5A,B). We compute the eigengenes of each module in turn and synthesize the closer modules into new ones. Finally, a total of nine modules were identified for subsequent analysis (Fig. 5C). The turquoise module was highly related to aneuploidy score (Fig. 5D). Thereby, a total of 1785 distinctly correlated module genes in this module were selected for further analysis.

### The establishment of an ARS model based on aneuploidy-related module genes and validation

**Figure 6:**
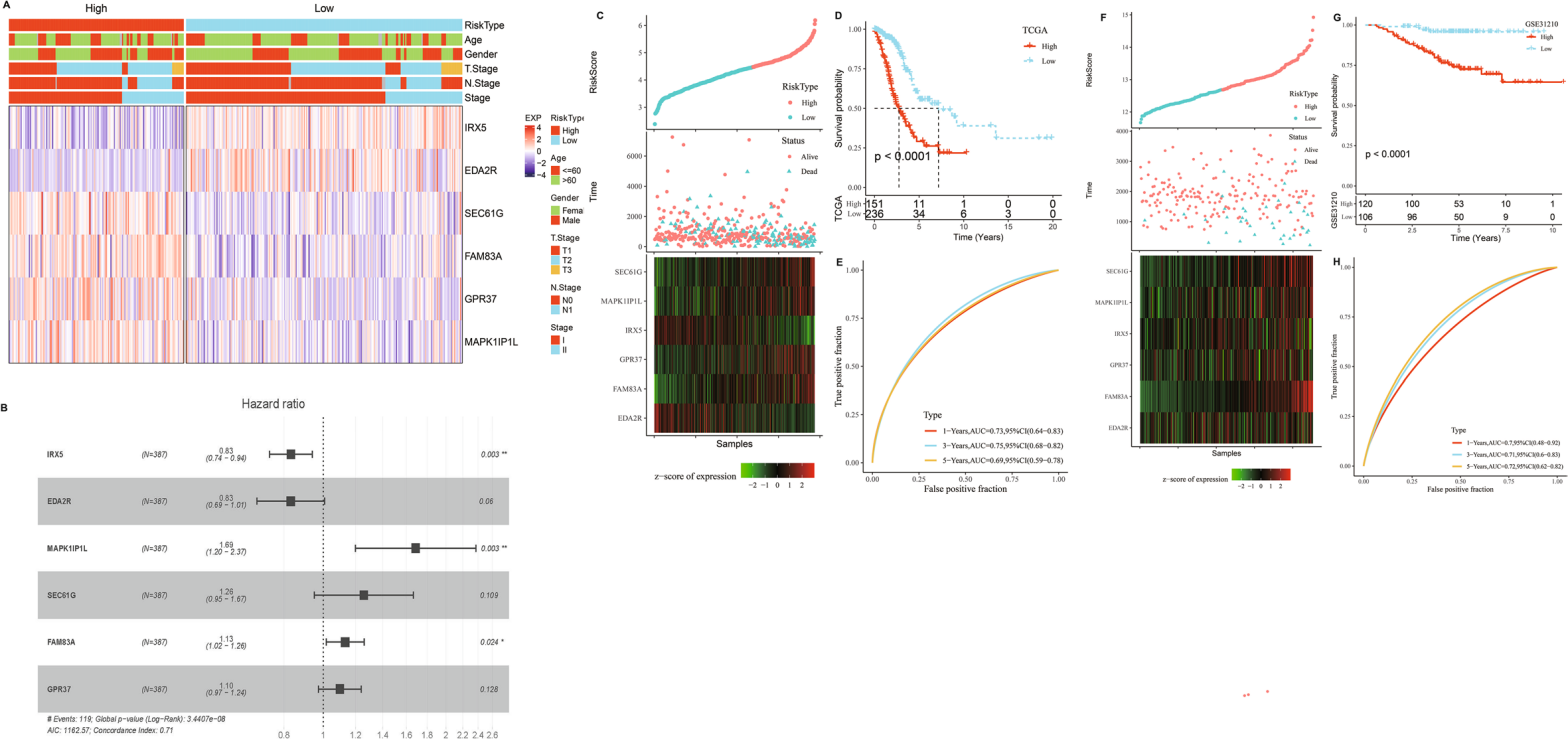
The establishment and assessment of a riskscore model. (**A**) The expression of 6 selected genes, distribution of Age, Gender, T. Stage, N. Stage and Stage in high and low risk groups. (**B**) Multivariate cox regression analysis of six selected genes. (**C**,**D**) Survival analysis between two risk groups in TCGA-LUAD cohort. (**E**) Time-ROC analysis between two risk groups in TCGA-LUAD cohort. (**F**–**H**) Model validation analysis in GSE41613 dataset.

As 1785 module genes were distinctly correlated with aneuploidy score, we first performed Univariate cox regression analysis and identified 116 genes closely connected to LUAD prognosis (*p* < 0.01, Supplementary Fig. 2A). 12 out of the 166 genes were preserved by LASSO-cox regression model with lambda at 0.0135 (Supplementary Fig. 2B,C). Further, through stepwise multivariate regression analysis, 6 genes were retained for establishing an ARS model. The detailed information of these genes was listed in Table 1. The expression levels of these 6 genes combining clinical features were displayed in Fig. 6A. In addition, we analyzed by multivariate cox regression to be used to further evaluate these 6 key genes (Fig. 6B). Each patient’s ARS was calculated based on the following formula (2):

2 $$ \begin{aligned} {\text{ARS}} & = - 0.183 \times {\text{Exp }}IRX5 - 0.184 \times {\text{Exp }}EDA2R \\ & \quad + 0.522 \times {\text{Exp }}MAPK1IP1L + 0.23 \times {\text{Exp }}SEC61G \\ & \quad + 0.124 \times {\text{Exp }}FAM83A + 0.094 \times {\text{Exp }}GPR37 \\ \end{aligned} $$

**Table 1:** Genes included for establishment of ARS model.

Gene name	Coefficient	Full name	Category
IRX5	− 0.183	Iroquois homeobox gene 5	Protein coding
EDA2R	0.184	Ectodysplasin A2 receptor	Protein coding
MAPK1IP1L	0.522	SEC61 translocon subunit‑gamma	Protein coding
SEC61G	0.23	Sec61 Translocon Gamma Subunit	Protein coding
FAM83A	0.124	Family with sequence similarity 83 member A	Protein coding
GPR37	0.094	G protein-coupled receptor 37	Protein coding

Correlation analysis between 6 key genes and genes affects aneuploidy showed a significant association (supplementary Fig. 3), indicating those genes closely correlated aneuploidy. Given optimal cutoff value, 151 patients were stratified into high ARS group, and 236 patients were separated into low ARS group. Patients with high ARS had worse survival status (even dead) and shorter survival time (Fig. 6C,D), indicating that samples with high ARS had poorer prognosis. Time-dependent ROC analysis validated the predictability of the ARS signature in LUAD as all values of area under the curve were higher than 0.6 (Fig. 6E). The model was also validated in GSE41613 dataset (Fig. 6F–H). In addition, as shown in Supplementary Fig. 4, there was a significant difference in the distribution of the three subtypes in the high and low ARS groups. We found that the proportion of C3 was the largest in the high ARS group, which was associated with its poorer prognosis.

### Construction and assessment of the nomogram

**Figure 7:**
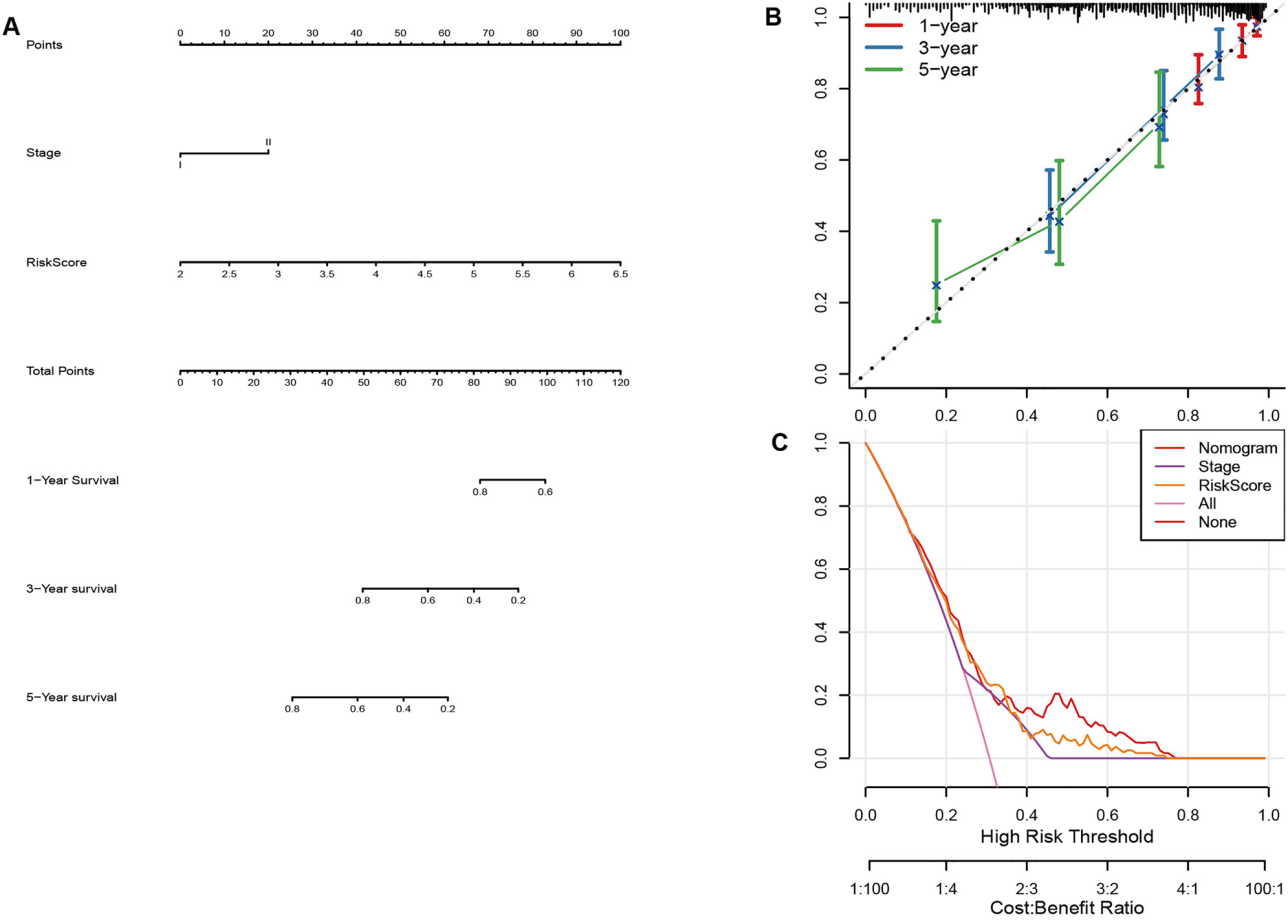
Nomogram analysis. (**A**) Design a nomogram. (**B**) The calibration curve. (**C**) The decision curve.

Univariate and Multivariate cox analysis showed only ARS and stage had prominent relevance to prognosis (Table 2). Therefore, a nomogram was designed ARS and stage. Figure 7A exhibited a liner chart to calculate survival rates of a patient. The total score was obtained through adding all the individual scores. The calibration curve showed favorable consistency between the predicted and ideal values of 1, 3, 5 years survival time (Fig. 7B). From the decision curve, both the nomogram and ARS had the optimal clinical net benefits (Fig. 7C). Briefly, the nomogram for LUAD had remarkable discrimination and calibration capacity.

**Table 2:** Univariate and multivariate cox analysis for validation the independence of ARS.

Variables	Univariate			Multivariate		
	HR (95%CI)	*P*	Significance	HR (95%CI)	*P*	Significance
ARS	2.7 (2–3.6)	8.8e−12	***	2.9 (2.1–3.9)	3.9e−12	***
Age	1.4 (0.93–2.1)	0.11				
Gender	1 (0.72–1.5)	0.85				
T.stage	1.4 (0.96–2.1)	0.079				
N.stage	2.1 (1.4–3.1)	0.00015	***	0.78 (0.42–1.4)	0.42	
Clinical stage	2.5 (1.7–3.6)	8.1e−07	***	3.1 (1.7–5.5)	0.00017	***

### Immunotherapy and drug sensitivity analysis applying ARS model

**Figure 8:**
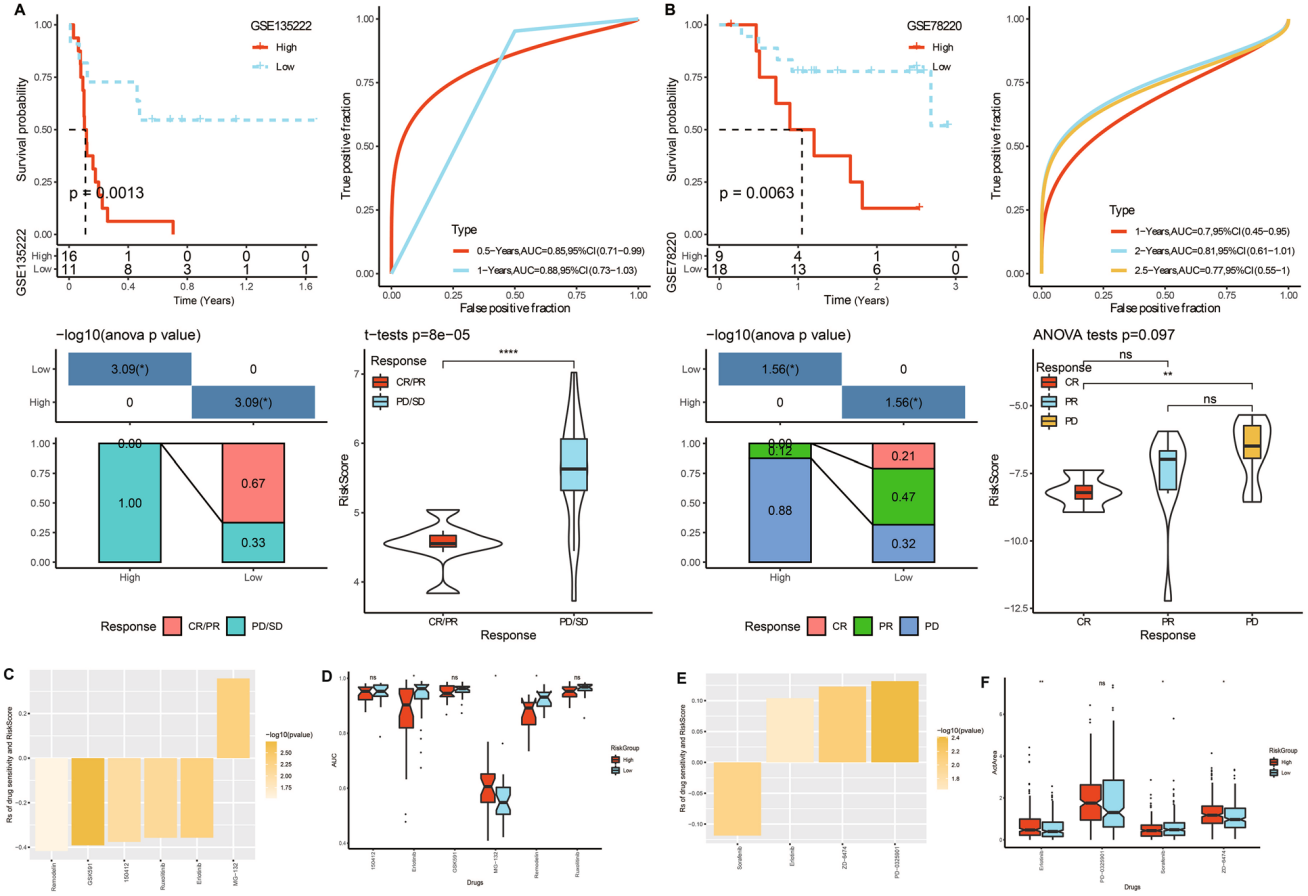
Immunotherapy and drug sensitivity analysis applying ARS model. (**A**) Immunotherapy evaluation in GSE135222 cohort. (**B**) Immunotherapy evaluation in GSE78220 cohort. (**C**,**D**) Drug sensitivity prediction using GDSC database. (**E**,**F**) Drug sensitivity prediction using CCLE database. *p < 0.05, **p < 0.01, ns: no significance.

Immune checkpoint inhibitors play a crucial role in cancer immunotherapy and has been widely adopted to treat multiple types of cancers^36^. *PD-1* and its ligand (*PD-L1*) are preferential therapeutic targets for immune checkpoint inhibitors^37,38^. We selected two immunotherapy datasets involving anti-PD-1 treatment to evaluate the potential of ARS model for immunotherapy. Based on our previously confirmed ARS formula and classifying method, patients treated by immunotherapy were successfully divided into high and low ARS groups. As seen in GSE135222 cohort, low ARS groups had prolonged survival time. Time-ROC analysis demonstrated the predictive capacity of the model. Higher proportions of progressive disease (PD)/stable disease (SD) were observed in high ARS group (Fig. 8A). Similar phenomenon was also detected in GSE78220 cohort with more PD patients in high ARS group (Fig. 8B). As for drug sensitivity prediction, high ARS patients were sensitive to MG-132, while low ARS patients were more sensitive to Erlotinib and Remodelin among 6 closely relevant medications selected from GDSC database (Fig. 8C,D). In another CCLE database, high ARS patients were sensitive to Erlotinib and ZD-6474, while low ARS patients were more sensitive to Sorafenib among 4 closely correlated medications (Fig. 8E,F).

## Discussion

The number of patients with LUAD is increasing significantly, and LUAD has been proven as the most prevalent subtype in lung cancers^39^. Along with in-depth investigations on cancer, aneuploidy involves point mutations, and whole-chromosome gains and losses as signs of cancer often occurs in an array of cancers^40,41^. Therefore, exploring aneuploidy relevant genes to evaluate the prognosis of patients with LUAD is meaningful. In this study, we firstly stratified TCGA-LUAD patients into 3 clusters based on CNV data with significant differences in the patterns of amplification and deletion in genomic regions. Given proper subgroup subtyping, we integrated intersecting DEGs and performed WGCNA and correlation analysis concerning aneuploidy to acquire significant hub module genes. Lasso analysis was then performed to build a 6-gene ARS model and a nomogram. Collectively, the ARS model contributed to the survival prediction for LUAD patients.

Reduced immune infiltration in high aneuploidy samples was observed within numerous cancer types^19^. Intensive work found that aneuploidy was irrelevant to the expression of immune signaling markers, positively correlated with genes of immune evasion, and could reduce response to immunotherapy^12,42,43^. Consistent with previous research, in the initial TCGA-LUAD grouping, C1 subgroup with a low degree of chromosomal CNV displayed favorable prognosis and high levels of immune infiltration. Patients in immunotherapy datasets were also divided into high and low ARS group based on the ARS model. Similarly, low ARS group patients displayed a distinct survival advantage and more active response to immunotherapy. Our work further supported the view that cancer aneuploidy could help predict patients’ survival after immunotherapy in future cancer therapy. More importantly, aneuploidy-related genes in specific cancer were expected to become drug research targets for cancer therapy.

A robust ARS model including 6 genes (*IRX5*, *EDA2R*, *MAPK1IP1L*, *SEC61G*, *FAM83A* and *GPR37*) was constructed. Cancer-related studies have enlightened the significance of these genes in tumorigenesis and pathogenesis. The Iroquois homeobox gene 5 (*IRX5*) facilitated metastasis of colorectal cancer cells via suppressing the RHOA-ROCK1-LIMK1 axis^44^. Another colorectal cancer study discovered that *IRX5* improved genomic instability in colorectal cancer cells as overexpressed *IRX5* decreased tumor cell proliferation and promoted G1/S cell cycle arrest and senescent activity^45^. In our research, up-regulated *IRX5* level was detected in high ARS group with poor prognosis, indicating an anticancer effect of *IRX5*. The possible mechanism should be analyzed in the future. *EDA2R* was a direct target of wild-type TP53. The enhanced expression of *EDA2R* in specimens may explain an unfavorable prognosis in ovarian cancer with wild-type TP53^46^. A reverse relationship between immune-related gene riskscore and *EDA2R* were also uncovered in other LUAD study^47^. Urine proteome profiling showed that high proportions of MAPK1IP1L could distinguish lung cancer patients from control and other cancers^47^. Sec61 Translocon Gamma Subunit (*SEC61G*) often played an oncogenic role through enhancing tumor cell proliferation^48^, metastasis^49,50^ and was negatively correlated with immune cell infiltration^51^. Therefore, the role of *SEC61G* was also studied in LUAD. Consistent with our finding, a high level of *SEC61G* was noticeably related to a poor prognosis in LUAD patients^52^. Family with sequence similarity 83 member A (*FAM83A*) was widely recognized as a oncogene, as it was frequently overexpressed in various tumors such as breast cancer^53^, ovarian cancer^54^ and cervical cancer cells^55^ or specimens with a poor prognosis. *FAM83A* was also reported to facilitate lung cancer development via wnt and hippo signaling pathways^56^. Wang H and colleagues discovered that regenerating islet-derived family, member 4, stimulated peritoneal metastasis in gastric cancer through G protein-coupled receptor 37 (*GPR37*)^57^. Xie et al. identified *GPR37* as a predictive biomarker for LUAD by obtaining LUAD differentially expressed genes from TCGA. They showed that *GPR37* was able to bind to *CDK6*, which in turn induced cell cycle arrest to promote tumor progression in LUAD^58^. These results suggest the importance of studying the potential relationship between aneuploidy-related gene and the prognosis of LUAD patients.

Furthermore, we found that patients in the LUAD high-risk group were more sensitive to MG-132. MG-132 as a proteasome inhibitor has been shown to be useful in the treatment of lung cancer patients^59^. Han et al.^60^ showed that.MG132 was able to inhibit the growth of Calu-6 lung cancer cells by promoting apoptosis and facilitating glutathione depletio. Remodelin is a small molecule inhibitor of N-acetyltransferase 10, which is thought to be able to reverse conditions of cancer development, including epithelial-mesenchymal transition, drug resistance and hypoxia^61^. In addition, Erlotinib in combination with signaling inhibitors (e.g., MK-2206) is also considered a potential advantage in the treatment of lung cancer^62^. In our study, patients in the low risk group of LUAD were more sensitive to Erlotinib and Remodelin. These results illustrate that a prognostic model based on aneuploidy-related genes can provide a good prediction of therapeutic agents for LUAD patients. Cancer prognostic models based on CNV-related genes have become a research hotspot for knowing tumor prognosis. Hu et al. developed a model to predict the prognosis of breast cancer patients based on CNV-related genes. The area of the ROC curve for this model was 0.7, 0.63, and 0.58 in the TCGA test set, while the AUC values were 0.66,0.68, and 0.71 in the TCGA all data sets^14^. In this study, we constructed a risk model based on aneuploidy-associated genes with AUC values of 0.7, 0.81, and 0.77 in the GEO cohort, respectively. This suggests that the predictive power of our constructed model is not inferior to that of previous studies. In order to facilitate further clinical application, we developed and calibrated a nomogram. The calibration curve showed that the nomogram was well calibrated. However, there were also some limitations to the clinical application of the model. Firstly, the signature of 6-gene was only developed using a TCGA cohort and validated in a GEO database. The nomogram was designed using only TCGA queue. In the future, we will use more LUAD cohorts to further calibrate nomogram for its clinical benefits.

## Conclusion

To sum up, our study illustrated that aneuploidy was closely connected to LUAD. Moreover, an ARS model generated based on 6 aneuploidy relevant genes could help predict LUAD patient’s survival, immunotherapy response and treatment selections to sensitive drugs. The present findings may offer a significant basis for future studies.

### Supplementary Information

Supplementary Legends.

Supplementary Figure 1.

Supplementary Figure 2.

Supplementary Figure 3.

Supplementary Figure 4.

Supplementary Table S1.

## Data Availability

The dataset used in this study is available in GSE31210 (https://www.ncbi.nlm.nih.gov/geo/query/acc.cgi?acc=GSE31210), GSE78220 (https://www.ncbi.nlm.nih.gov/geo/query/acc.cgi?acc=GSE78220), GSE135222 (https://www.ncbi.nlm.nih.gov/geo/query/acc.cgi?acc=GSE135222), GSE41613 (https://www.ncbi.nlm.nih.gov/geo/query/acc.cgi?acc=GSE41613), GSE78220 (https://www.ncbi.nlm.nih.gov/geo/query/acc.cgi?acc=GSE78220).

## Abbreviations

ARS: Aneuploidy related riskscore
AUC: Area under concentration–time curve
CCLE: Cancer Cell Line Encyclopedia
CDF: Cumulative distribution function
CNV: Copy Number Variation
DC: Dendritic cells
DEGs: Differentially expressed genes
ESTIMATE: Estimation of STromal and Immune cells in MAlignant Tumor tissues using Expression data
FDR: False discovery rate
GEO: Gene Expression Omnibus
GDSC: Genomics of Drug Sensitibity in Cancer
LASSO: Least absolute shrinkage and selection operator
LUAD: Lung adenocarcinoma
PD: Progressive disease
PD-1: Programmed cell death 1
ROC: Receiver operating characteristic analysis
SD: Stable disease
TCGA: The Cancer Genome Atlas
WGCNA: Weighted correlation network analysis
