# Supplementary material for: An original aneuploidy-related gene model for predicting lung adenocarcinoma survival and guiding therapy
Source: Sci Rep. 2024 Apr 7;14:8135. doi: 10.1038/s41598-024-58020-y (PMC10999435; doi:10.1038/s41598-024-58020-y)
Supplement: Supplementary file 1 — Supplementary Legends. [file 41598_2024_58020_MOESM1_ESM.docx]

**Supplementary Figure 1:** Functional enrichment analysis of DEGs in clusters. (A) Functional enrichment analysis of DEGs between Cluster1 and Cluster3. (A) Functional enrichment analysis of DEGs between Cluster1 and Cluster2. (A) Functional enrichment analysis of DEGs between Cluster2 and Cluster3.

**Supplementary Figure 2:** The establishment of a riskscore model based on aneuploidy related module genes. (A) Univariate Cox regression analysis for prognostic gene based on significant module genes. (B-C) LASSO-cox regression analysis.

**Supplementary Figure 3:** Correlation analysis between genes in RisScore and aneuploidy related genes.

**Supplementary Figure 4:** Distribution of the three LUAD subtypes in the high and low ARS groups.
