# Supplementary figures and images for: An original aneuploidy-related gene model for predicting lung adenocarcinoma survival and guiding therapy
Source: Sci Rep. 2024 Apr 7;14:8135. doi: 10.1038/s41598-024-58020-y (PMC10999435; doi:10.1038/s41598-024-58020-y)

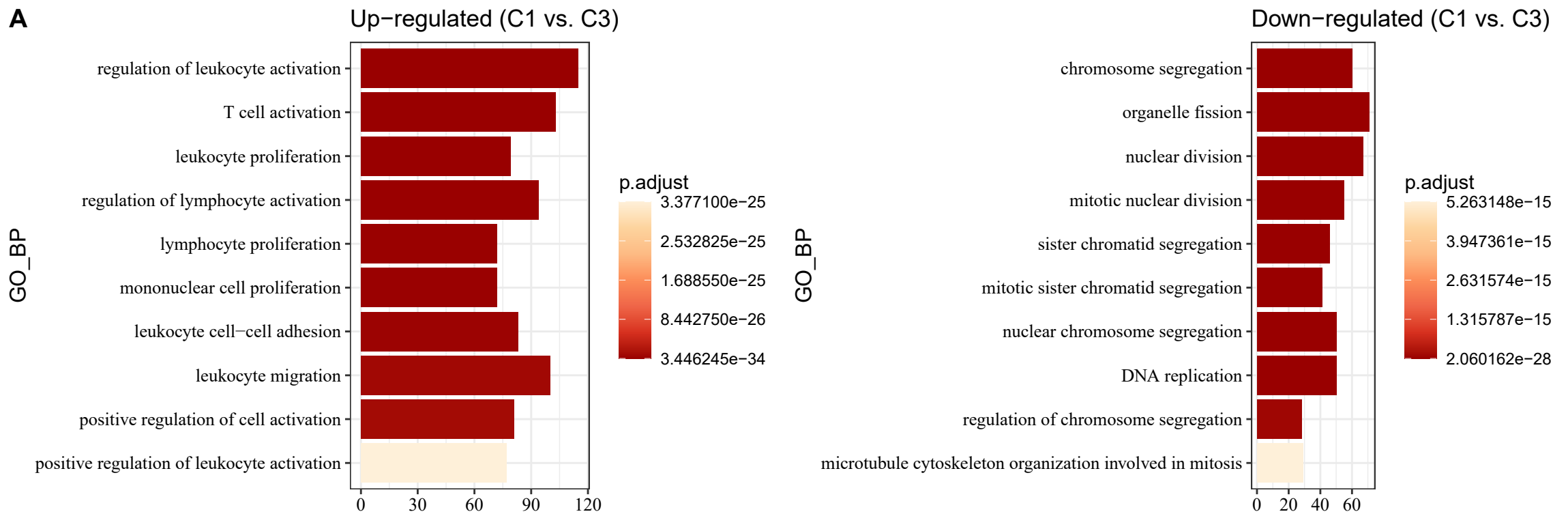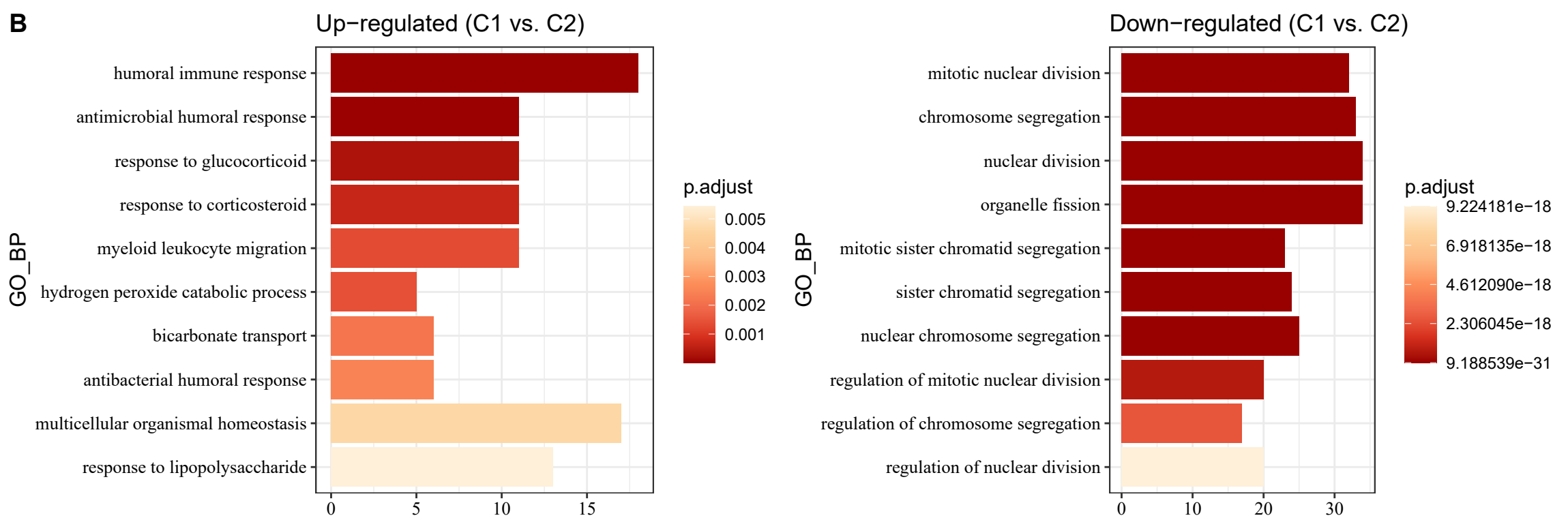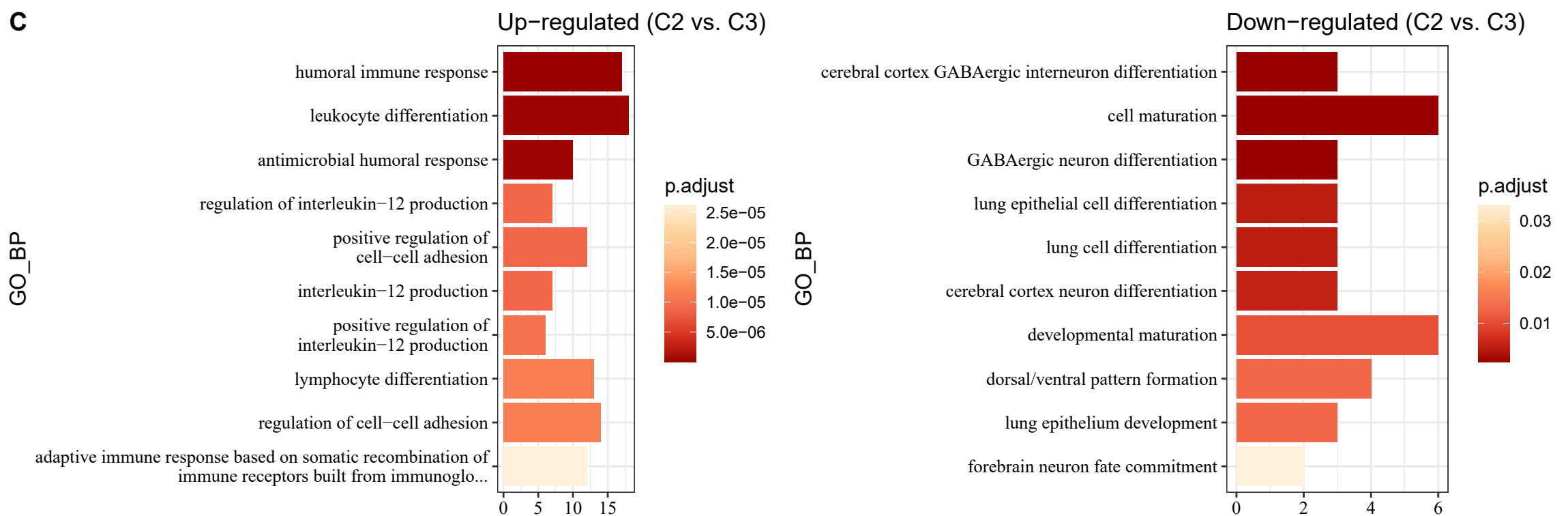

Supplement: Supplementary file 2 — Supplementary Figure 1. [file 41598_2024_58020_MOESM2_ESM.pdf]

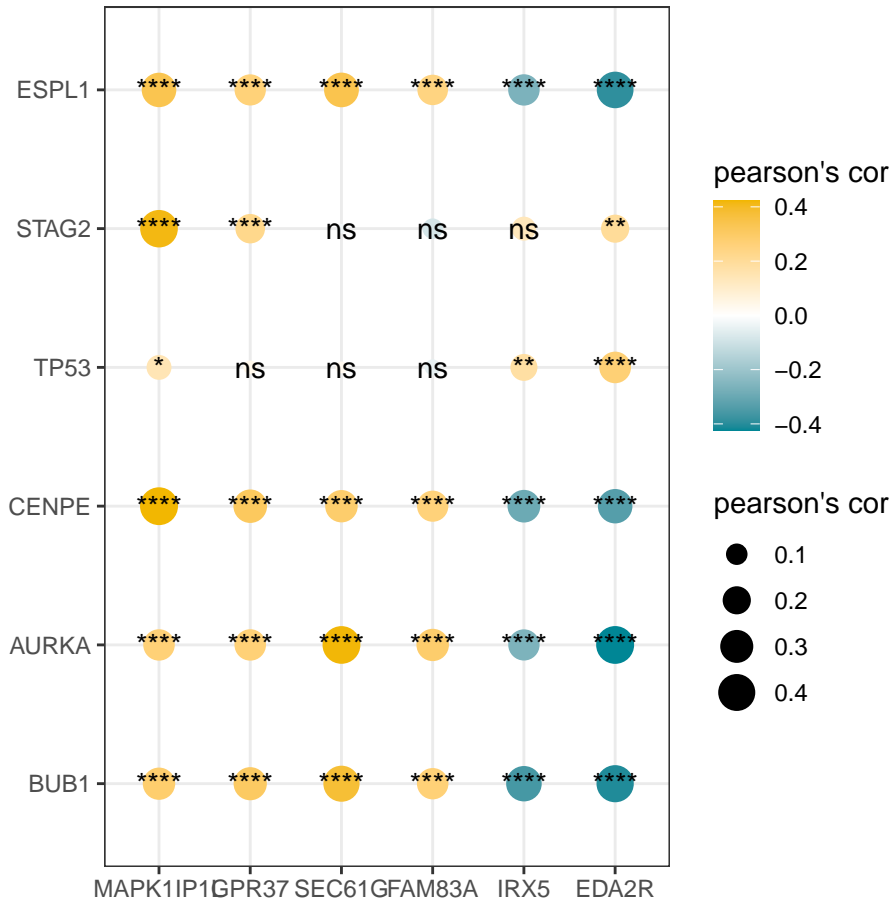

Supplement: Supplementary file 4 — Supplementary Figure 3. [file 41598_2024_58020_MOESM4_ESM.pdf]

$-\log_{10}(\text{chisq.test p value})$

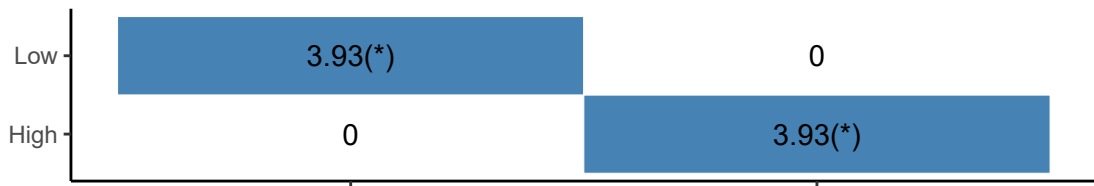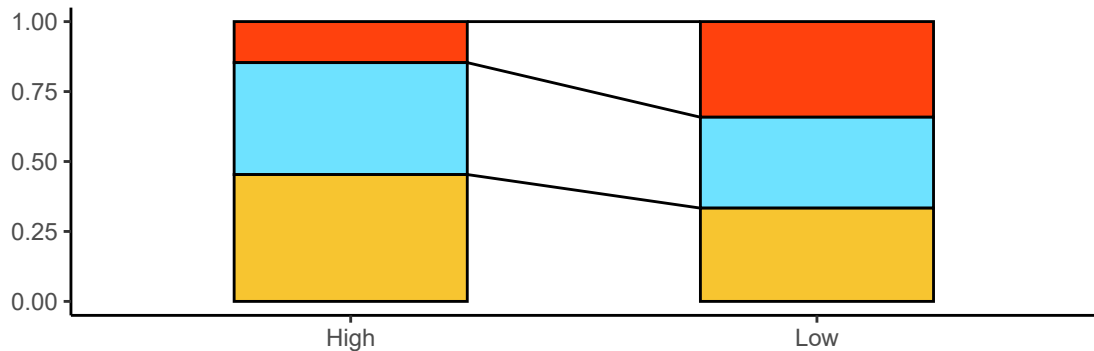

Cluster ■ C1 ■ C2 ■ C3

Supplement: Supplementary file 5 — Supplementary Figure 4. [file 41598_2024_58020_MOESM5_ESM.pdf]
