# Supplementary material for: An original aneuploidy-related gene model for predicting lung adenocarcinoma survival and guiding therapy
Source: Sci Rep. 2024 Apr 7;14:8135. doi: 10.1038/s41598-024-58020-y (PMC10999435; doi:10.1038/s41598-024-58020-y)

A

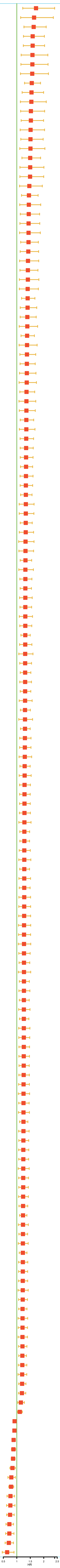

Genes	p.value	Hazard Ratio(95% CI)
PSMC6	1.8e-03	1.71(1.22,2.4)
PSMD12	7.2e-03	1.64(1.14,2.36)
SEC61G	1.5e-04	1.63(1.27,2.09)
LDHA	1.7e-04	1.59(1.25,2.02)
GNPNAT1	2.2e-04	1.59(1.24,2.03)
RSRC1	3.6e-03	1.58(1.16,2.15)
YKT6	4.3e-03	1.58(1.15,2.16)
MAPK1IP1L	6.1e-03	1.57(1.14,2.17)
ERO1A	4.6e-06	1.56(1.29,1.88)
SMS	8.1e-04	1.55(1.2,2)
C11orf24	5.7e-03	1.54(1.13,2.09)
MTHFD1	4.2e-03	1.52(1.14,2.03)
PSMD2	2.3e-03	1.52(1.16,1.99)
EIF3B	6.0e-03	1.51(1.13,2.03)
C17orf58	3.0e-03	1.51(1.15,1.98)
POFUT1	8.2e-03	1.51(1.11,2.04)
RIPK2	5.5e-04	1.5(1.19,1.88)
YWHAG	7.0e-03	1.5(1.12,2.01)
PSMD11	7.2e-03	1.49(1.11,2)
FKBP3	8.4e-03	1.47(1.1,1.95)
CCT6A	5.2e-04	1.45(1.18,1.79)
FKBP9	6.4e-03	1.45(1.11,1.89)
H2AFZ	3.4e-03	1.44(1.13,1.85)
GPI	5.0e-03	1.44(1.12,1.85)
LRIF1	7.4e-03	1.44(1.1,1.87)
CENPH	1.8e-03	1.44(1.14,1.8)
SPDL1	3.4e-03	1.43(1.13,1.81)
PVR	6.1e-03	1.42(1.1,1.81)
P4HA1	3.9e-03	1.41(1.12,1.78)
DHFR	7.7e-03	1.41(1.1,1.82)
ALYREF	7.7e-03	1.4(1.09,1.8)
E2F7	1.6e-04	1.4(1.18,1.67)
H2AFX	2.3e-03	1.4(1.13,1.74)
FEN1	2.2e-03	1.39(1.13,1.72)
FAAP24	8.0e-03	1.39(1.09,1.77)
KPNA2	3.8e-04	1.38(1.16,1.65)
RANBP1	8.4e-03	1.38(1.09,1.75)
POLR3G	3.0e-03	1.38(1.12,1.7)
KIF18A	2.2e-03	1.37(1.12,1.68)
FKBP4	4.8e-03	1.37(1.1,1.71)
PAICS	8.2e-03	1.37(1.08,1.73)
BLM	2.7e-03	1.36(1.11,1.66)
SMC2	8.4e-03	1.36(1.08,1.7)
KRAS	5.9e-03	1.36(1.09,1.69)
KNL1	1.2e-03	1.35(1.13,1.63)
CDCA4	5.2e-03	1.35(1.09,1.67)
SHCBP1	1.4e-03	1.35(1.12,1.62)
WDHD1	1.1e-03	1.35(1.13,1.61)
CDC25C	9.7e-04	1.34(1.13,1.6)
OIP5	5.1e-04	1.34(1.14,1.59)
DTL	1.3e-03	1.34(1.12,1.6)
SPC25	9.6e-04	1.34(1.13,1.59)
TK1	3.1e-04	1.34(1.14,1.57)
CCDC34	5.6e-03	1.34(1.09,1.64)
SGO2	5.2e-03	1.33(1.09,1.63)
ECT2	7.2e-04	1.33(1.13,1.57)
DIAPH3	4.6e-03	1.33(1.09,1.62)
LAMC1	9.7e-03	1.32(1.07,1.64)
TICRR	7.4e-03	1.32(1.08,1.62)
HMMR	6.5e-04	1.32(1.13,1.55)
CHAF1B	7.8e-03	1.32(1.08,1.62)
TYMS	1.3e-03	1.32(1.11,1.56)
CCNB1	6.2e-04	1.32(1.13,1.54)
CHEK1	2.3e-03	1.32(1.1,1.57)
PRC1	9.9e-04	1.32(1.12,1.55)
TACC3	3.6e-03	1.32(1.09,1.58)
ARHGAP11A	1.4e-03	1.32(1.11,1.56)
ANLN	4.0e-05	1.32(1.15,1.5)
CDCA2	1.9e-03	1.31(1.11,1.56)
NCAPD2	7.6e-03	1.31(1.07,1.6)
RHPN2	1.4e-03	1.31(1.11,1.55)
PLK1	4.0e-04	1.31(1.13,1.52)
CENPU	1.4e-03	1.31(1.11,1.54)
CDCA5	4.0e-04	1.31(1.13,1.52)
RAD51	4.1e-03	1.31(1.09,1.57)
RRM2	2.2e-04	1.31(1.13,1.5)
FANCI	7.3e-03	1.31(1.07,1.59)
DLGAP5	1.5e-04	1.3(1.14,1.5)
KIF20A	1.2e-03	1.3(1.11,1.53)
CKAP2L	1.5e-03	1.3(1.11,1.53)
CENPM	4.4e-03	1.3(1.08,1.55)
CDKN3	6.0e-04	1.29(1.12,1.5)
PTTG1	3.3e-03	1.29(1.09,1.53)
KIF14	1.4e-03	1.29(1.1,1.51)
EXO1	1.2e-03	1.29(1.1,1.5)
DEPDC1B	1.4e-03	1.29(1.1,1.5)
CCNB2	1.9e-03	1.28(1.1,1.5)
SGO1	5.3e-03	1.28(1.08,1.53)
CCNA2	6.3e-04	1.28(1.11,1.48)
HJURP	5.3e-04	1.28(1.11,1.47)
FAM111B	2.7e-03	1.28(1.09,1.5)
ASF1B	5.6e-03	1.28(1.07,1.52)
KIF4A	6.8e-04	1.28(1.11,1.47)
CENPE	4.5e-03	1.28(1.08,1.51)
KIF23	2.4e-03	1.28(1.09,1.49)
SKA3	4.7e-03	1.28(1.08,1.51)
GPRIN1	5.4e-03	1.27(1.07,1.51)
UBE2S	6.1e-03	1.27(1.07,1.51)
CENPK	8.3e-03	1.27(1.06,1.52)
RACGAP1	7.7e-03	1.27(1.07,1.51)
CENPI	9.7e-03	1.27(1.06,1.52)
C17orf53	5.1e-03	1.27(1.07,1.5)
TESMIN	3.0e-03	1.27(1.08,1.48)
CKS1B	9.8e-03	1.27(1.06,1.52)
NDC80	1.5e-03	1.27(1.09,1.47)
MAD2L1	4.0e-03	1.27(1.08,1.49)
ASPM	1.5e-03	1.26(1.09,1.46)
PKMYT1	5.6e-03	1.26(1.07,1.49)
NEK2	9.6e-04	1.26(1.1,1.45)
PCLAF	5.6e-03	1.26(1.07,1.49)
NUSAP1	4.6e-03	1.26(1.07,1.48)
AURKA	3.4e-03	1.26(1.08,1.47)
SKA1	3.6e-03	1.26(1.08,1.47)
CDK1	2.7e-03	1.26(1.08,1.46)
TMEM171	3.8e-03	1.26(1.08,1.47)
PFKP	5.8e-03	1.25(1.07,1.47)
UHRF1	8.3e-03	1.25(1.06,1.48)
HMGA1	4.8e-03	1.25(1.07,1.46)
CLSPN	7.4e-03	1.25(1.06,1.47)
TPX2	4.4e-04	1.25(1.1,1.41)
GTSE1	5.3e-03	1.25(1.07,1.46)
DEPDC1	3.6e-03	1.24(1.07,1.44)
PRR11	5.0e-03	1.24(1.07,1.45)
BUB1B	4.1e-03	1.24(1.07,1.44)
KIF11	9.9e-03	1.24(1.05,1.46)
CEP55	4.6e-03	1.24(1.07,1.44)
NCAPG	3.3e-03	1.24(1.07,1.43)
PIMREG	4.8e-03	1.24(1.07,1.43)
BIRC5	1.9e-03	1.23(1.08,1.41)
FAM83A	2.3e-04	1.23(1.1,1.38)
BUB1	6.2e-03	1.23(1.06,1.43)
FOXM1	2.1e-03	1.23(1.08,1.4)
MCM10	7.2e-03	1.23(1.06,1.42)
CTSV	6.4e-04	1.23(1.09,1.38)
CDC45	4.2e-03	1.23(1.07,1.41)
CENPF	3.8e-03	1.22(1.07,1.4)
KIFC1	4.6e-03	1.22(1.06,1.4)
ESPL1	9.7e-03	1.22(1.05,1.42)
MELK	3.7e-03	1.22(1.07,1.4)
SLC2A1	2.8e-03	1.21(1.07,1.38)
FAM83D	9.3e-03	1.21(1.05,1.4)
MKI67	7.5e-03	1.21(1.05,1.4)
NCAPH	8.5e-03	1.21(1.05,1.4)
CDC20	3.5e-03	1.21(1.06,1.38)
GPR37	2.0e-03	1.21(1.07,1.36)
IL1R2	4.9e-03	1.21(1.06,1.37)
KIF2C	7.9e-03	1.2(1.05,1.37)
IGF2BP3	1.7e-03	1.2(1.07,1.34)
UBE2C	2.4e-03	1.19(1.06,1.33)
MYBL2	9.5e-03	1.15(1.04,1.28)
TNNT1	9.0e-03	1.11(1.03,1.21)
NAPSA	7.2e-03	0.92(0.86,0.98)
SFTPB	1.2e-03	0.91(0.86,0.96)
CYP4B1	1.0e-03	0.88(0.82,0.95)
CACNA2D2	3.7e-03	0.88(0.8,0.96)
NKX2_1	7.1e-04	0.86(0.79,0.94)
TMEM163	4.5e-03	0.84(0.75,0.95)
VIPR1	9.3e-03	0.8(0.67,0.95)
IRX5	6.0e-05	0.79(0.7,0.89)
EDA2R	3.9e-03	0.76(0.64,0.92)
ACOXL	8.1e-03	0.76(0.62,0.93)
ALDH2	2.3e-03	0.75(0.62,0.9)
GNG7	1.0e-03	0.73(0.6,0.88)
KLHDC8B	2.8e-03	0.72(0.59,0.89)
IVD	1.7e-03	0.7(0.57,0.88)
ZFP2	9.2e-03	0.64(0.46,0.9)

B

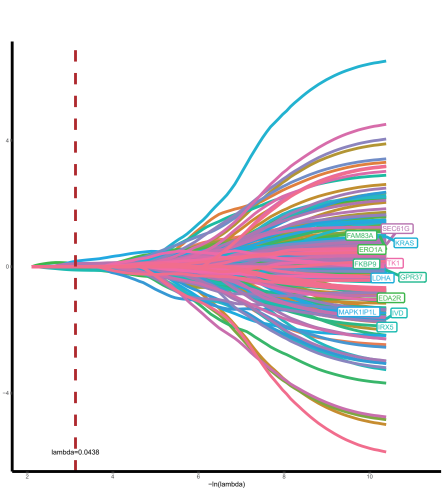

C

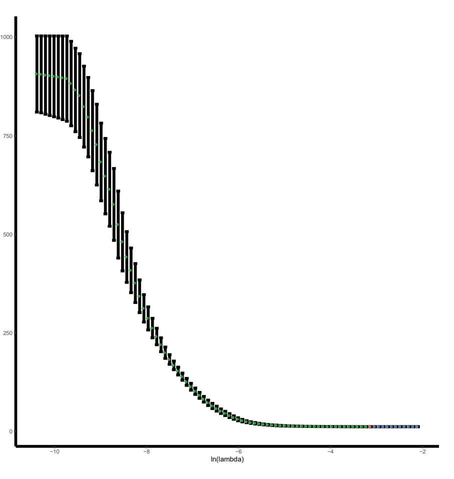

Supplement: Supplementary file 3 — Supplementary Figure 2. [file 41598_2024_58020_MOESM3_ESM.pdf]
